# An exploration of eating behaviours and caregiver mealtime actions of children with Tourette syndrome

**DOI:** 10.3389/fped.2022.933154

**Published:** 2022-09-07

**Authors:** Bobbie L. Smith, Amanda K. Ludlow

**Affiliations:** Department of Psychology, Sports Science and Geography, School of Life and Medical Sciences, University of Hertfordshire, Hatfield, United Kingdom

**Keywords:** food avoidant, food approach, Autism Spectrum Disorder (ASD), Attention Deficit Hyperactivity Disorder (ADHD), food selectivity, Tourette syndrome (TS), emotional eating, neurodiversity

## Abstract

Food avoidant behaviours are common concerns amongst individuals with Tourette syndrome, with high levels of food selectivity reported in children and food neophobia and avoidant restrictive eating behaviours in adults. However, less is known about food approach behaviours. The current study aimed to explore differences in food approach and food avoidant eating behaviours in children with Tourette syndrome (TS) and their relationship to caregiver mealtime actions. Thirty-seven caregivers of children with Tourette syndrome were compared with children with Autism Spectrum Disorders, children with Attention-Deficit/Hyperactivity Disorder and a control group. Caregivers completed the Child Eating Behaviour Questionnaire and Parent Mealtime Action Scale-Revised. Caregiver-reported findings revealed that children with Tourette syndrome exhibited more food approach behaviours, specifically greater food responsiveness, emotional overeating and desire to drink, compared to controls. Children from the three neurodiverse groups had similar levels of emotional overeating and food selectivity, which were all significantly higher than the control group. Positive persuasion was uniquely identified as a mealtime strategy adopted by caregivers of children with Tourette syndrome. The results suggest that children with Tourette syndrome are at more risk of showing a broader array of food difficulties than previously reported, including food avoidant and approach behaviours. It is encouraged that clinicians monitor eating behaviour in appointments with children with Tourette syndrome.

## Introduction

Eating behaviour research has developed two concepts which broadly describe movements towards or away from food consumption, named food approach and food avoidant behaviours, respectively. Food avoidant behaviours include food selectivity (also known as food fussiness), the rejection of familiar and novel food, slowness in eating, emotional undereating and regulating eating through internal cues, namely satiety responsiveness (as characterised by the Child Eating Behaviour Questionnaire) (1). In contrast, food approach behaviours encompass movements and desires towards food, which can be characterised by emotional overeating, desire to drink and responses to external stimuli, including enjoyment of food and food responsiveness. Within the literature, emotional over- and undereating have been defined as the consumption of more or less food than is considered to be within typical eating patterns and is largely considered as a stress response an individual experiencing unwanted feelings. Moreover, food responsiveness is related to overeating as individuals are heavily influenced to eat in response to external cues, such as sight and smell. This eating behaviour is in contrast to satiety responsiveness, whereby the individual responds to internal cues of fullness to cease consumption. Collectively, increased food responsiveness and emotional overeating mean that children eat when they are not necessarily hungry. A reduced ability to regulate mechanisms related to hunger decreases with age (2) and has been found to have adverse consequences in terms of weight, nutritional intake and subsequent health complications. Since children with Tourette syndrome (TS) have been shown to differ in their response to food [e.g., (3)], the present study investigates food approach and food avoidant behaviours in children with and without TS, in comparison to children with Autism Spectrum Disorders (ASD) and Attention-Deficit/Hyperactive Disorder (ADHD), to understand the eating profiles, but also to address children's eating in relation to caregiver mealtime actions.

Recently, research has explored food avoidant behaviours in individuals with TS, a neurodevelopmental disorder characterised by involuntary, repetitive and non-rhythmic motor and phonic tics (4). Food avoidant behaviours were found to be common concerns amongst individuals with TS (3), with higher levels of food selectivity reported in children outside of the normative period of 6 years of age (5). Moreover, there was evidence to suggest that anomalous eating behaviours are present in adulthood, with higher levels of food selectivity, food neophobia and avoidant/restrictive eating behaviours reported in adults with TS compared to individuals without (3, 6). Research has also shown similar levels of heightened food selectivity in children with TS and two other neurodevelopmental disorders, namely ASD and ADHD, even when accounting for comorbidity in comparison to children showing typical development (7). This finding weakens the argument that additional comorbid diagnoses may underlie increased food selectivity found in TS.

To date, the literature has focused solely on food avoidant behaviours in TS. However, to provide context to the breadth of eating challenges and wider eating profile of children with TS, food approach behaviours must also be considered. Food selectivity appears to be a transdiagnostic characteristic of neurodivergent children with disordered eating found to be widespread in children with a diagnosis of ASD or ADHD (8). Despite these similarities, research has also suggested that children with ASD and ADHD seem to differ in their overall eating profiles. For example, children with ASD show greater food avoidant behaviours compared to controls, including heightened food selectivity and emotional undereating (9). Children with ASD have also been shown to have obsessive eating routines (10) whereas, binge, hedonic and emotional overeating have been found to influence the positive association between symptoms of ADHD and BMI (11).

Some similarities in levels of food selectivity may be partially accounted for by the heightened sensory sensitivity, a commonly reported symptom in neurodevelopmental disorders (5). For example, sensory-focused eating is frequently reported in neurodiverse children leading to a limited diet (10). In contrast, some differences indicated within the literature may be better explained by impulsivity, a core symptom of ADHD, which has been related to uncontrolled eating behaviour, weight gain and bulimia nervosa symptoms (12). Symptoms specific to TS include tics, which have not been previously evidenced to influence eating behaviours, instead have been thought to be influenced by nutritional intake. Many individuals with TS also describe an aversive and unpleasant internal urge that precipitates the release of a tic, known as a premonitory urge (13). Whilst up to 93% of patients report experiencing a premonitory urge, this characteristic is not currently included in the DSM-5 diagnostic criteria. Given that premonitory urges are sensory phenomena and previous research has correlated sensory processing with eating behaviours, such urges may be associated with eating behaviours in individuals with TS. Overall, considering the adverse consequences in terms of weight and diet quality for some eating behaviours and their prevalence in children within neurodiversity research, exploring the wider eating profiles of children with TS and how they compare to comorbid disorders, is needed.

In addition to the health consequences of maladaptive eating behaviours, the literature consistently reports that these behaviours can be disruptive to mealtimes leading to increased parental stress, issues maintaining routines and inappropriate mealtime interactions (14). For example, caregivers' mealtime behaviours have been widely associated with eating behaviours, particularly of young children showing typical patterns of development. During the early years, caregivers have greater control over mealtimes in terms of when food is presented, what foods are offered, and their quantities. Furthermore, research has suggested strategies that the caregiver can use to provide an effective environment and deal with challenging eating patterns to encourage the development of healthy eating behaviours. Modelling, for example, can be a positive strategy to improve acceptance of fruit and vegetables (15). In contrast, parental strategies, including pressure to eat and high levels of control are counterproductive by encouraging maladaptive eating patterns. For example, an authoritarian and restrictive parenting style has been associated with increased emotional eating in children (16). These strategies have also been associated with a reduced ability of the child to regulate their energy intake, and a paradoxical interest in forbidden foods which are more commonly used in children who are considered overweight (17).

While a large volume of literature emphasises the role of the caregiver in influencing their child's eating behaviour, it is increasingly being evidenced in both qualitative and quantitative research that there is a bidirectional relationship between caregiver mealtime behaviours and child eating behaviours (18, 19). A complex interplay of variables influences a child's eating behaviour with both the child and caregiver having agency to contribute to the mealtime experience. In the ASD literature, research has shown caregivers are more likely to encourage and prompt eating compared to controls which aligns with typical caregiver responses to their children exhibiting food avoidant behaviours (9). Furthermore, caregivers have been found to prepare more special meals for children with ASD compared to children without (20).

In comparison to other neurodiverse populations, there is currently an absence of research focusing on the role of the caregiver at mealtimes for children with TS. However, this is particularly important to address for children with TS given food avoidance previously demonstrated differences in caregiver responses to food refusal. More specifically to TS, anecdotal evidence has suggested that children with TS show a predisposition to higher weight status, with a national survey in Iran indicating the greatest prevalence of tic disorders was in males who were either overweight or obese (21). Additional research has suggested that a diagnosis of TS increases the risk of having obesity and is associated with a significant risk of cardiometabolic disorders (22, 23). Furthermore, the medication used to treat TS, such as neuroleptic drugs, make individuals particularly vulnerable to weight gain (24). Moreover, longitudinal work has shown that a child with a heavier weight status predicts later use of controlling feeding practises, suggesting a possible cyclic relationship with weight status as a mediating factor.

The current study was exploratory in nature and its purpose was 3-fold: (1) to explore any differences in food approach, and food avoidant behaviours in TS compared to a control group, (2) to explore further any of these differences in eating behaviours compared to other commonly occurring neurodiverse children, namely those with ASD or ADHD and (3) to explore relationships between child eating behaviours and caregiver mealtime behaviours. It was hypothesised that similar to previous findings differences in avoidant eating behaviours, namely food selectivity, would be found between children with TS and controls, but no significant differences between the ADHD and ASD groups. Similar to the findings that ASD and ADHD show more differences from each other in their food approach behaviours, the TS group was expected to show more differences across this domain when comparing the neurodiverse conditions (3). Given the relationship established between food selectivity and caregiver mealtime behaviours, it was expected to be a relationship between caregivers' actions and children with TS eating behaviours.

## Materials and methods

### Participants

One hundred and twenty-four caregivers [22–67 y; *M* (*SD*) = 41(8) y], 118 mothers and four fathers and two legal guardians (grandmother), completed the online survey. One hundred and five caregivers described their nationality as British, one as French, six as Canadian, one as Maltese, one as Italian and eight as American. Caregivers were asked to confirm whether their child had a clinical diagnosis of a neurodevelopmental disorder. Authors are aware of the comorbidity between neurodevelopmental disorders and therefore, only children with a sole clinical diagnosis of one of the three disorders focused on in this study were included. Of the responses, 37 children had a diagnosis of TS (6 females, 31 males) and were between the ages of 6 years 7 months and 15 years 0 months. The Premonitory Urge for Tics Scale (PUTS) (25) was completed by caregivers alongside their children, only in the TS group. A score above 31 indicates extremely high intensity with probable severe impairments. In the current sample scores ranged from 9 to 36 (*M* = 22.54, *SD* = 5.97). Of the children with TS diagnosis taking medication (*n* = *15*), the most commonly reported was melatonin (*n* = 8). Other prescription drugs recorded were sertraline (*n* = 4) and clonidine (*n* = 3).

The comparison groups included a control group, children with ASD and children with ADHD. The control group comprised 36 children without a clinical diagnosis of a neurodevelopmental disorder between the ages of 6 and 16 years (13 females, 23 males). Caregivers of children with ASD completed the Autism Spectrum Screening Questionnaire (26); all children reached the cut-off scores (*M* = 25.87, *SD* = 10.12). Thirty-six children between the ages of 6 and 17 years with a clinical diagnosis of ASD were included in the current study. Finally, twenty children with a clinical diagnosis of ADHD (8 females, 12 males) between the ages of 6 and 16 years were included in the current study. All children in this group met the required T-score of 65 or above on the Connors' Parent Rating Scale-Revised (27). The groups did not differ in age, *F*_(3, 114)_ = 1.88, *p* = 0.138.

### Measures

Participants provided background information about their age, ethnicity, height, and weight, as well as their child's sex, date of birth, height and weight and any clinical diagnosis including comorbid disorders. Participants were able to enter their child's weight anthropometric measurements in the format most convenient and the researchers later converted the measurements according to the metric system. All caregivers were then asked to complete the following two standardised questionnaires:

#### The children's eating behaviour questionnaire (CEBQ: 5)

The CEBQ is a 35-item measure designed to identify the frequency of a child's eating behaviour on eight independent scales, which can be grouped into two subsets of eating behaviour. Firstly, the food approach eating profile which is the average of four subscales encompasses a desire to carry drinks on their person (desire to drink), eating as a response to external stimuli (food responsiveness & enjoyment of food) and over-eating as an emotional response to negative feelings (emotional overeating). Secondly, the food avoidant eating profile which is the average of four subscales measuring the ability to regulate eating through internal cues (satiety responsiveness), slowness in eating, reducing food consumption as an emotional reaction to negative feelings (emotional undereating) and rejecting a large amount of novel and familiar foods (food fussiness). 'Food selectivity' was the chosen term for the current study to highlight the behaviours exist outside of the normative developmental period as well as reflecting severity of consequences of such behaviours. As the eight subscales are independent and can be additionally grouped to categorise eating behaviours, they were treated as separate when running the statistical analysis, meaning no adjustments were used. Caregivers rated the frequency with which their child exhibits the behaviour on a 5-point Likert scale ranging from 1 (never) to 5 (always). Development of the questionnaire revealed good internal reliability coefficients (Cronbach's alpha) for all the subscales, ranging from 0.74 to 0.91 (4). The Cronbach alpha for the present study ranges between 0.63 and 0.96.

#### Parent mealtime action scale-revised (PMAS-R; 35)

The PMAS-R is a 31-item questionnaire with the following nine subscales: setting snack limits, using positive persuasion, insistence on eating, fat reduction techniques and use of rewards during mealtimes, providing daily fruit and vegetable availability, showing snack modelling, making children special meals different from the family meal, and allowing too many food choices. Caregivers rated how often they exhibited these mealtime behaviours on a 5-point Likert scale ranging from 1 (never) to 5 (always). The mean internal reliability of Cronbach alpha is 0.66 and the mean test-retest reliability score of 0.71 (22). The scale was developed with a non-clinical sample, however, has been consistently used within and validated in a clinical sample (22) and there is a mean internal reliability Cronbach alpha of 0.68 in the current study.

### Procedure

The research was granted ethical approval from the University of Hertfordshire Ethical Advisory Committee, Protocol Number: aLMS/PGT/UH/02784(4), and the research was performed in accordance with the Declaration of Helsinki. Participants were recruited through Tourettes Action charity, online forums and local organisations that agreed to advertise the study. A survey link was provided for participants to learn about the study *via* an online participant information sheet which provided further details. If after reading the information sheet, participants wished to take part in this research, participants were first required to give informed consent by signing an online consent form before progressing to the survey. The questionnaires were presented in the same order to each participant and took ~25 min to complete. The questionnaire remained active for 2 months. Families were provided with no financial incentive to take part. At the end of the study, participants were provided information with sources of support for any concerns around their child's eating behaviours.

### Data analysis

Firstly, BMI z-scores (BMIz) for children were calculated using the Child Growth Foundation's (28) growth references which adjust for age and sex. Standard definitions for thinness, overweight and obesity corrected for age and sex were used to categorise children's BMI (kg/m^2^; 22). Standard definitions for thinness, overweight and obesity corrected for age and gender were used to categorise children's BMI (29, 30). Data analysis was conducted in IBM SPSS Statistics (RRID: SCR_016479). A One-way ANOVA was conducted to compare differences in BMIz between the four groups. Secondly, two-tailed Pearson's correlations were used to establish whether child age and sex were related to food approach and food avoidant eating behaviours. Thirdly, to examine whether there were differences in food approach, food avoidant and caregiver feeding behaviours between children with TS and the control group a series of independent *t*-tests were conducted on all subscales of the CEBQ. Fourthly, One-way ANOVAs were conducted to explore differences in all subscales of the CEBQ and PMAS-R between the four groups. Finally, two-tailed Pearson's correlations were conducted to analyse the relationship between food avoidant and approach behaviours, BMIz, and caregiver mealtime behaviours.

## Results

### Participant characteristics

Outside of the main questionnaires, there was a small subset from each of the four groups who chose to complete the current weight and height of their child. Nineteen caregivers of children with TS, 18 caregivers of children without a clinical disorder (control group) and 16 caregivers in ADHD and ASD groups provided this information. There was no significant difference in BMIz scores between the four groups, *F*_(3, 66)_ = 1.667, *p* = 0.183. Of the TS sample who provided child BMI data (*n* = 27), 29.6% were categorised as underweight. More specifically, 7.4% were categorised as grade 2 (a BMI below 17) and 22.2% were categorised as grade 3 (a BMI below 16). Moreover, 22.2% of children in the TS group were categorised as overweight, and 14.8% were classified as obese. Of the controls who provided child BMI data (*n* = 27), 25.9% were categorised as underweight [grade 1 (a BMI below 18.5) = 3.7%, grade 2 = 11.1%, grade 3 = 11.1%], 11.1% (*n* = 3) were overweight and 3.7% were classified as obese. Although a Pearson chi-square test revealed no significant difference in the number of children categorised as a healthy compared to unhealthy weight status between each of the groups, *X*^2^(1, *N* = 54) = 3.650, *p* = 0.056, significantly more children with TS were categorised as being overweight and obese compared to the control group, *X*^2^ (1, *N* = 73) = 4.51, *p* = 0.034.

The data was then analysed to establish whether the children's age or sex were related to their food approach and food avoidant behaviours. An independent samples *t*-test revealed no significant difference in food approach, *t*_(122)_ = 0.435, *p* = 0.664, and food avoidant behaviours, *t*_(122)_ = −0.1.009, *p* = 0.315, between males and females when comparing the total sample of children. Therefore, sex was not controlled for in further analyses.

Two-tailed Pearson's correlations revealed a positive relationship between food approach behaviours and age, *r*_(37)_ = 0.67, *p* < 0.001, and a negative relationship between food avoidant behaviours and age, *r*_(37)_ = −0.74, *p* = 0.001, in children with TS. These findings suggest younger children showed a different pattern than older children. No significant correlations between age, food approach and food avoidant behaviours were identified for the control group, children with ASD or children with ADHD (*p* > 0.05). Child demographic information and descriptive statistics for all standardised measures are shown in Table 1.

**Table 1:** Results of One-way ANOVAs, and Tukey's HSD *post hoc* tests, for eating behaviours between the children with Tourette syndrome, Autism Spectrum Disorder, Attention Deficit Hyperactive Disorder or controls.

	**Mean (SD)**						
	**TS (*n* = 37)**	**CG (*n* = 36)**	**ASD (*n* = 31)**	**ADHD (*n* = 20)**	***F*** **_(3, 120)_**	***p***	**Tukey's HSD**
**Demographics**							
Age (y)	10.15 (2.64)	9.09 (2.44)	10.43 (3.32)	10.41 (3.59)			
Height (cm)	146.22 (18.01)	140.44 (14.57)	145.43 (26.92)	142.24 (22.05)			
Weight (kg)	38.23 (17.22)	36.27 (17.02)	42.72 (19.66)	52.60 (15.64)			
BMIz	0.57 (4.12)	−0.89 (1.78)	0.87 (1.36)	0.82 (2.14)			
**CEBQ**							
*Food approach*	3.12 (0.97)	2.59 (0.48)	3.10 (0.74)	3.01 (0.99)	2.54	0.060	–
Desire to drink	2.81 (1.28)	2.17 (0.72)	2.69 (1.14)	2.43 (1.12)	2.41	0.071	–
Enjoyment	3.52 (1.29)	3.74 (0.66)	2.47 (1.15)	3.60 (0.94)	0.45	0.719	–
Food responsiveness	3.32 (1.32)	2.48 (0.76)	2.84 (1.20)	3.18 (1.42)	3.54	0.017	TS > CG
Emotional overeating	2.71 (1.07)	1.92 (0.68)	2.61 (1.00)	2.83 (1.11)	5.83	0.001	TS > CG, ASD > CG, ADHD > CG
*Food avoidant*	2.87 (0.79)	2.66 (0.59)	2.90 (0.89)	2.86 (0.74)	2.07	0.108	–
Emotional undereating	2.91 (0.78)	2.48 (0.76)	3.26 (0.86)	3.00 (0.83)	5.11	0.002	ASD > CG
Food selectivity	3.42 (1.27)	2.76 (0.87)	3.74 (1.03)	3.37 (1.01)	4.95	0.003	TS > CG, ASD > CG, ADHD > CG
Satiety responsiveness	2.68 (1.08)	2.75 (0.75)	2.80 (0.90)	2.59 (0.76)	0.27	0.849	–
Slowness in eating	2.39 (1.26)	2.63 (0.82)	2.59 (0.97)	2.50 (1.07)	0.39	0.763	–

TS, Tourette syndrome; CG, Control Group; ASD, Autism Spectrum Disorders; ADHD, Attention-Deficit/Hyperactive Disorder; BMIz, Body Mass Index z-score; CEBQ, Child Eating Behaviour Questionnaire.

### What were the eating behaviours of children with TS compared to controls?

Independent *t*-tests were conducted to examine whether there were group differences in food avoidant and approach behaviours between children with TS and the controls. As shown in Table 2, children with TS show greater food approach behaviours than the control group, more specifically greater food responsiveness, emotional overeating and desire to drink. There was no significant difference in the overall food avoidant eating profile between the two groups. However, of the food avoidant subscales children with TS scored significantly higher on food selectivity and emotional undereating than the controls.

**Table 2:** Independent t-tests exploring differences in eating behaviours between children with TS and the control group.

	***df***	***t***	***p***
*Food approach*	53	−2.92	0.005
Desire to drink	57	−2.62	0.01
Enjoyment	54	0.92	0.36
Emotional overeating	61	−3.72	<0.001
Food responsiveness	58	−3.34	<0.001
*Food avoidant*	71	−1.26	0.21
Emotional undereating	71	−2.29	0.03
Food selectivity	64	−2.56	0.01
Satiety responsiveness	64	0.31	0.77
Slowness in eating	62	0.99	0.32

### How did the eating behaviours of children with TS compare to children with ASD or ADHD?

One-way ANOVAs were conducted to identify differences between food approach and food avoidant eating behaviours between the four groups. As shown in Table 1, significant differences were found in food selectivity, food responsiveness and emotional over- and under-eating. *Post hoc* Tukey's HSD tests revealed that children with TS had similar levels of food selectivity and emotional overeating compared to children with ASD and children with ADHD. All three clinical groups had a significantly higher tendency of emotional overeating and food selectivity compared to children showing typical development. Higher levels of food responsiveness were found in children with TS compared to the controls, whereas children with ASD had higher levels of emotional undereating compared to the controls. No other significant differences in eating behaviours were found between the groups.

### What were the relationships between eating behaviours, caregiver mealtime actions and BMIz?

One-way ANOVAs were run to examine differences in caregiver mealtime behaviours, as measured across eight subscales of the PMAS-R between children with TS, children with ASD, children with ADHD and the control group (see Table 3). Caregivers of children with TS reported using insistence and positive persuasion less compared to the caregivers of the control group. Some differences were also observed in caregiver mealtime actions for children with ASD, including reduced availability of fruit and vegetables compared to controls and more special meals compared to caregivers of children with ADHD.

**Table 3:** Results of one-way ANOVAs, and Tukey's HSD *post hoc* tests, for caregiver mealtime actions between the children with Tourette syndrome, Autism Spectrum Disorder, Attention Deficit Hyperactive Disorder or controls.

	**Mean (SD)**						
	**TS (*n* = 37)**	**CG (*n* = 36)**	**ASD (*n* = 31)**	**ADHD (*n* = 20)**	***F*** **_(3, 120)_**	***p***	**Tukey's HSD**
**PMAS-R**							
Snack limits	4.00 (1.28)	4.01 (0.95)	3.69 (1.06)	4.02 (1.00)	0.66	0.581	–
Daily fruit & –vegetables	4.25 (0.76)	4.57 (0.69)	3.84 (1.21)	4.05 (1.36)	3.22	0.025	ASD < CG
Positive persuasion	3.74 (0.71)	3.79 (0.69)	2.88 (1.34)	3.03 (1.10)	7.45	<0.001	TS <ASD, TS < ADHD, ASD < CG, ADHD < CG
Use of rewards	2.56 (0.80)	2.75 (0.76)	2.54 (0.86)	2.50 (0.91)	0.61	0.607	–
Insistence	1.88 (0.97)	2.61 (0.92)	1.71 (0.61)	2.35 (1.04)	7.17	<0.001	TS < CG, ASD < CG, ASD > ADHD
Snack modelling	2.34 (0.81)	2.09 (0.65)	2.04 (0.82)	2.33 (0.89)	1.25	0.297	–
Special meals	2.29 (0.67)	2.66 (0.70)	2.71 (0.70)	2.08 (0.85)	4.80	0.003	ADHD < CG, ADHD < ASD
Fat reduction techniques	3.16 (0.95)	2.66 (0.87)	2.68 (0.99)	2.95 (0.99)	2.27	0.084	–
Many food choices	3.02 (0.66)	2.63 (0.51)	2.73 (1.00)	2.75 (0.91)	1.77	0.157	–

TS, Tourette syndrome; CG, Control Group; ASD, Autism Spectrum Disorders; ADHD, Attention-Deficit/Hyperactive Disorder; PMAS-R, Parent Mealtime Action Scale-Revised.

A series of Two-tailed Pearson's correlations were conducted to examine associations between the eating behaviours found to be significantly different among the four groups and caregiver mealtime actions (results are shown in Table 4). When subsequent partial correlations were conducted to control for age, no significant correlations between any of the subscales were found. Caregiver and child behaviours were subsequently explored in relation to child BMIz scores. Two-tailed Pearson's correlations revealed that emotional overeating was positively associated with BMIz in the TS group, *r*_(20)_ = 0.55, *p* = 0.012. There were no other significant correlations between BMIz and eating behaviours in any of the four groups. Regarding BMIz and caregiver mealtime actions, a negative correlation was identified with positive persuasion in the control group; a positive correlation with many special meals in the ASD group and a positive correlation with fat reduction techniques in the ADHD group. It is important to note that all significant correlations with caregiver mealtime actions were no longer significant when controlling for age (*p* > 0.05).

**Table 4:** Two-tailed Pearson's correlations between parent mealtime action subscales and child eating behaviours.

	**Snack limits**	**Daily fruit & vegetables availability**	**Positive persuasion**	**Use of rewards**	**Insistence**	**Snack modelling**	**Special meals**	**Fat reduction techniques**	**Many food choices**
**CG**									
*Food approach*	0.27	−0.15	−0.14	0.31	0.21	0.07	0.35^*^	0.35^*^	0.27
Emotional overeating	0.17	−0.33^*^	−0.17	0.29	0.01	−0.13	0.32	−0.06	0.59^***^
Food responsiveness	0.27	−0.09	−0.13	0.25	0.30	0.03	0.39^*^	0.43^**^	0.28
*Food avoidant*	−0.023	−0.23	−0.13	0.11	0.04	0.02	0.20	−0.16	0.50^**^
Food selectivity	−0.03	−0.33^*^	−0.17	0.07	−0.08	0.02	0.34^*^	−0.14	0.53^***^
Emotional undereating	0.04	0.003	−0.07	0.32	0.06	−0.06	0.35^*^	−0.07	0.48^**^
BMIz	0.08	−0.10	−0.61^**^	−0.34	−0.19	0.24	−0.19	−0.17	−0.05
**TS**									
*Food approach*	0.18	0.03	−0.11	−0.25	−0.40^*^	−0.21	−0.49^**^	0.57^***^	−0.31
Emotional overeating	0.11	0.18	−0.07	−0.18	−0.24	−0.07	−0.53^***^	0.51^***^	−0.32^*^
Food responsiveness	0.20	−0.04	−0.06	−0.09	−0.33^*^	−0.13	−0.47^**^	0.56^***^	−0.28
*Food avoidant*	−0.42^**^	−0.10	0.40^*^	0.22	0.31	0.006	0.45^**^	−0.41^**^	0.41^*^
Food selectivity	−0.47^**^	−0.11	0.38^*^	0.12	0.23	−0.08	0.55^***^	−0.22	0.34^*^
Emotional undereating	−0.19	0.01	0.31	0.34^*^	0.26	−0.14	0.31	−0.09	0.28
BMIz	−0.14	0.07	0.35	0.32	0.13	−0.08	−0.38	0.27	−0.13
**ASD**									
*Food approach*	−0.03	0.13	0.08	−0.05	−0.10	0.20	−0.08	0.10	−0.03
Emotional overeating	0.17	0.23	0.00	0.05	−0.28	0.18	−0.08	0.27	0.17
Food responsiveness	0.00	0.02	0.07	−0.01	−0.11	0.12	−0.02	0.07	0.00
*Food avoidant*	0.19	−0.07	0.11	0.09	0.02	−0.14	0.06	0.16	0.19
Food selectivity	0.12	0.00	0.29	0.36^*^	0.02	0.08	0.17	0.27	0.12
Emotional undereating	0.29	−0.04	0.15	0.01	−0.24	−0.17	−0.01	0.19	0.29
BMIz	−0.08	−0.31	0.44	0.03	0.23	0.29	0.58^*^	0.35	−0.06
**ADHD**									
*Food approach*	0.09	0.38	0.33	0.24	0.13	0.57^**^	0.04	0.43	0.09
Emotional overeating	0.09	0.38	0.39	0.16	0.06	0.51^*^	0.01	0.33	0.09
Food responsiveness	0.08	0.29	0.29	0.29	0.14	0.55^*^	0.00	0.32	0.08
*Food avoidant*	0.13	−0.13	−0.08	−0.02	0.10	−0.35	0.07	−0.11	0.13
Food selectivity	−0.14	−0.24	−0.06	−0.08	−0.03	−0.38	−0.06	−0.08	−0.14
Emotional undereating	0.24	0.03	−0.01	0.00	−0.11	−0.21	0.12	0.01	0.24
BMIz	−0.14	−0.07	−0.18	0.25	0.40	0.09	−0.14	0.59^*^	0.32

BMIz in children with TS, Tourette syndrome; ASD, Autism Spectrum Disorder; ADHD, Attention-Deficit Hyperactive Disorder; or the CG, control group.

*** *p* ≤ 0.001;

** *p* < 0.01;

* *p* < 0.05. All correlations were no longer significant when controlling for age.

### Are there relationships between premintory urges and eating behaviours?

Two-tailed Pearson's correlations revealed that the PUTS was not correlated with any subscale of the CEBQ or the PMAS-R (*p* < 0.05). There was a significant correlation between BMIz and premonitory urges, *r*_(19)_ = 0.57, *p* = 0.01 suggesting those with more premonitory urges had higher BMIz, this relationship remained significant even when controlling for age, *r*_(18)_ = 0.37, *p* = 0.012. The child's age was not significantly associated with tic severity, *r*_(31)_ = 0.22, *p* = 0.230. In children with TS, both emotional overeating and tic severity was positively related to BMIz. Therefore, a multiple linear regression was carried out with both as predictors of BMIz. This revealed a significant model, *R*^2^ = 0.49, *F*_(2, 18)_ = 7.59, *MSE* = 78.63, *p* = 0.005, with both being found to be independent predictors (emotion overeating, β = 0.42, *t* = 2.28, *p* = 0.036; PUTS, β = 0.46, *t* = 2.50, *p* = 0.024). Children with TS who were reported as having more premonitory urges and/or were more emotional eaters had higher levels of BMIz.

## Discussion

The current study explored the eating behaviours of children with TS in comparison to a control group and how their profile compares to children with a diagnosis of ASD or ADHD. Caregiver-reported findings revealed that children with TS exhibited more food approach behaviours, specifically greater food responsiveness, emotional overeating and desire to drink, compared to controls. While the overall profile of food avoidance was not found to be significantly different between groups, children with TS did display significantly higher levels of food selectivity and emotional undereating compared to the controls. When comparing eating behaviours with other neurodiverse populations, similarities in food selectivity and emotional overeating were identified in all three neurodiverse groups. Importantly, children with TS who exhibited higher emotional overeating appeared more at risk of having a BMIz. The current study also identified differences in caregiver mealtime actions and some associations between child and caregiver behaviours in all four groups; however, this was no longer significant when controlling for age.

Differences in eating behaviours between children with TS and the control group were identified. Firstly, consistent with previous research, children with TS showed heightened food fussiness (3). Secondly, similar to research on children with ADHD (31, 32), greater desire to drink, emotional overeating and food responsiveness were identified in children with TS. Increased food responsiveness and emotional under- and overeating are related to eating based on external cues, meaning children could eat when they are not necessarily hungry. Infants' innate ability to regulate food intake (2) decreases with age (33), resulting in greater influence from external stimuli in the development of eating behaviours. A reduced ability to regulate mechanisms related to hunger has been found to have adverse consequences in terms of weight.

Similar to previous research [e.g., (34)], it was found that increased BMIz was associated with greater food approach behaviours, specifically increased emotional overeating was associated with higher BMIz in children with TS only. Emotional overeating refers to the consumption of food as a response to feeling negative emotions; therefore, the individual may eat when they are not hungry which can lead to greater consumption of food and therefore weight gain. Eating in response to emotions may reflect a reduced ability to self-regulate their appetite (35) and deficits in the emotional regulation (36). Therefore, research has suggested that interventions for emotional eating should focus on stress reduction techniques and the promotion of positive mood (37). These findings are particularly pertinent as research has indicated that there is a greater prevalence of anxiety disorders in children with tic disorders (38).

While there was no significant difference between the BMIz scores between the groups, the weight classification of children with TS was noteworthy. Regarding weight classification, children with TS fell at the two polar ends of the weight categories with 66% of children classified as having an unhealthy weight status, and more children were categorised as overweight or obese compared to the control group. This finding is aligned with the prevalence of psychiatric disorders being higher among children and adolescents who are overweight, specifically research found the most prevalence of tic disorders in males with overweight or obesity (21). While exploring the role of medication on BMI was outside the scope of the current study, it is important that future research explores this factor as some medications can be appetite-suppressing (e.g., ADHD treatment) whereas others can lead to weight gain (e.g., neuroleptics) (22). Overall, it is clinically important for clinicians to monitor weight and address any eating concerns, particularly for children displaying emotional overeating.

Premonitory urges are uncomfortable physical sensations preceding tics and are considered an important predictor of tic severity, even when controlling for age (39). The current study failed to establish a relationship between PUTS and any of the subscales of CEBQ or PMAS-R, potentially suggesting that severity of tics not to be a predictor of eating behaviours. Importantly, this measure has been identified as one of five recommended instruments for severity of tics. However, it is more reflective of the sensory phenomena associated with tics and may be more suitable for those of 10 years and older. Therefore, one of the major limitations of the current study is the lack of inclusion of a tic severity and frequency measure, such as the YGTSS a self-report measure that indicates clinically relevant exacerbations of tics, or The Proxy Report Questionnaire for Parents and Teachers, which has also been identified as a highly promising tool [for a full review of tic measure see (40)]. A tic frequency and severity questionnaire is important to include in future research to be able to establish whether those with more intense and severe motor and/or vocal tics show more disturbance in eating behaviours.

It was important to compare the eating behaviours of children with TS with other neurodiverse populations with no overlapping comorbidities to explore the argument of whether eating behaviours in TS can simply be explained by the underlying effects of ASD or ADHD. Given that similar levels of emotional overeating and food selectivity were identified across the three clinical groups, this shows that comorbidity does not explain these maladaptive patterns. Nevertheless, the authors do acknowledge that due to the comorbidities between the three clinical disorders and despite no comorbid diagnoses at the time of the study, there may be some overlap and later diagnoses to follow meaning the groups may not have been completely distinct. However, some differences in eating behaviours were identified. While the ASD and ADHD groups showed no significant differences in comparison to the controls, the TS group was unique in showing significantly higher food responsivity. These findings demonstrate that whilst neurodiverse populations do share symptomology, diagnoses and some eating behaviours, there are some distinctive eating behaviours related specifically to TS. Ultimately, clinicians need to monitor and ask about any eating concerns even when the child is presenting with a sole diagnosis of TS. Further to this, it is widely acknowledged that TS is comorbid with anxiety disorders, such as Obsessive-Compulsive Disorders (41), so it would be important for future research to establish how these disorders contribute to the eating profile of individuals with TS.

The role of the caregiver in children's eating behaviours was also investigated in the current study. There were differences in caregiver mealtime techniques between the four groups. Caregivers of children with TS reported the use of more insistence and positive persuasion compared to the three clinical groups. Caregiver mealtime behaviours were not associated with BMI, which is agreeable with research on children with ASD (42) and typically developing children (43). Food approach behaviours were negatively associated with special meals and fat reduction techniques, and the inverse was found for overall food avoidance in children with TS. Fat reduction techniques are used by caregivers of children with healthy diets, but also by caregivers of children who are overweight (44). These findings highlight the complex and multi-directional nature of caregiver and child mealtime behaviours.

In terms of special meals, it is common that caregivers to stop reoffering a given food after only three to five failed attempts and begin to make special meals (45). Meals separate from the family often include palatable high-calorie foods which are more likely to be accepted. This technique can be useful to increase weight if the child is underweight, however for children with food fussiness this technique can maintain and perpetuate the child's restricted diet (44). As food selectivity is especially common in children with TS, guidance to promote effective strategies for caregivers is needed. One caveat is that when controlling for the age of the child, the relationships were no longer significant between caregiver and child mealtime behaviours in the current study. This suggests that children are less influenced by their caregivers as they begin to make their own choices, and other factors may become more influential. Taken together, it is important to educate caregivers on effective strategies, such as repeated exposure, especially in their child's younger years. Early interventions are particularly relevant as eating behaviours established in childhood can continue into adolescents and adulthood (46).

One limitation of the current study is its cross-sectional design, which inhibits conclusions of causal relationships between caregiver mealtime behaviours and child eating behaviours. Longitudinal research is needed to draw conclusions about the bidirectional child-caregiver association in relation to eating behaviours taking into consideration any parental neurodevelopmental and co-morbidities (47). In addition, there was also missing data for the BMIz, and caregiver-reported anthropometrics may have led to a miscalculation of BMI. While objective measures are ideal, research has demonstrated a high level of accuracy in caregiver-reported height and weight measurements when compared with objective measurements (48). The data was collected *via* caregiver-report meaning there may be some social desirability on completion of the caregiver mealtime action measure. Perhaps the use of observations may prove useful in future research to explore the caregiver-child interaction during mealtimes to provide an insight which may be missed when through self-report (49). Understanding the eating behaviours of children with TS and factors which can influence these behaviours is clinically relevant for the development of effective interventions (50). The current study has demonstrated the adverse effect of increased emotional overeating on BMI, but research has also shown that eating behaviours can have an adverse impact on nutrient intake, which needs requires further investigation.

While the current study focused specifically on comparing TS with ADHD and ASD. It is important to note that TS has many underlying comorbidities. For example, a large proportion of TS patients meet a concurrent diagnosis for OCD (30–50%) (51). Furthermore, elevated rates of tics symptomology (10–30%) have also been reported in OCD patients (52, 53). To address eating difficulties associated with tics and/or TS, future studies will need to screen for co-morbidities such as anxiety disorders to understanding their role on eating behaviours in children with TS. Similarly, assessing for sensory processing disorders and their severity would help understand their role in eating behaviours.

Overall, this research identified that children with TS have a different eating profile to children with typical development, specifically heightened food approach behaviours, with implications of heightened emotional overeating increasing BMI status. Caregiver mealtime behaviours, specifically fat reduction and special meal techniques were associated with food approach and food avoidant eating behaviours. However, this relationship was more prominent in younger children. It is encouraged that clinicians monitor eating behaviour and BMI status in appointments with children with TS.

## Data availability statement

The raw data supporting the conclusions of this article will be made available by the authors, without undue reservation.

## Ethics statement

The studies involving human participants were reviewed and approved by University of Hertfordshire University Ethical Advisory Committee, Protocol Number: aLMS/PGT/UH/02784(4). The participants provided their written informed consent to participate in this study.

## Author contributions

AL and BS contributed to the conception and design of the study. BS performed the statistical analysis and wrote the first draught of the manuscript. AL wrote sections of the manuscript. All authors contributed to manuscript revision, read, and approved the submitted version.

## Funding

APC was funded by the University of Hertfordshire.

## Conflict of interest

The authors declare that the research was conducted in the absence of any commercial or financial relationships that could be construed as a potential conflict of interest.

## Publisher's note

All claims expressed in this article are solely those of the authors and do not necessarily represent those of their affiliated organizations, or those of the publisher, the editors and the reviewers. Any product that may be evaluated in this article, or claim that may be made by its manufacturer, is not guaranteed or endorsed by the publisher.

## References

[1] Wardle J Guthrie CA Sanderson S Rapoport L. Development of the children's eating behaviour questionnaire - wardle - 2001 -. J Child Psychol Psychiatry. (2001) 42:963–70. 10.1111/1469-7610.00792 11693591

[2] Birch LL Deysher M. Conditioned and unconditioned caloric compensation: evidence for self-regulation of food intake in young children. Learn Motiv. (1985) 16:341–55. 10.1016/0023-9690(85)90020-7

[3] Smith B Rogers SL Blissett J Ludlow AK. The role of sensory sensitivity in predicting food selectivity and food preferences in children with Tourette syndrome. Appetite. (2019) 135:131–6. 10.1016/j.appet.2019.01.003 30634006

[4] American Psychiatric Association. Diagnostic and Statistical Manual of Mental Disorders. 5th ed. (2013).

[5] Cano SC Tiemeier H Hoeken DV Tharner A Jaddoe VWV Hofman A. Trajectories of picky eating during childhood: a general population study. Int J Eat Disord. (2015) 48:570–9. 10.1002/eat.22384 25644130

[6] Smith BL Gutierrez R Ludlow AK, A. comparison of food avoidant behaviours and sensory sensitivity in adults with and without Tourette syndrome. Appetite. (2022) 168:105713. 10.1016/j.appet.2021.105713 34563498

[7] Smith B Rogers SL Blissett J Ludlow AK. The relationship between sensory sensitivity, food fussiness and food preferences in children with neurodevelopmental disorders. Appetite. (2020) 150:104643. 10.1016/j.appet.2020.104643 32105808

[8] Råstam M Täljemark J Tajnia A Lundström S Gustafsson P Lichtenstein P. Eating problems and overlap with ADHD and autism spectrum disorders in a nationwide twin study of 9- and 12-year-old children. Sci World J. (2013) 2013: 315429. 10.1155/2013/315429 23690743 PMC3654255

[9] Kral TVE Souders MC Tompkins VH Remiker AM Eriksen WT Pinto-Martin JA. Child eating behaviors and caregiver feeding practices in children with autism spectrum disorders. Public Health Nurs Boston Mass. (2015) 32:488–97. 10.1111/phn.12146 25112438

[10] Aponte CA Romanczyk RG. Assessment of feeding problems in children with autism spectrum disorder. Res Autism Spectr Disord. (2016) 21:61–72. 10.1016/j.rasd.2015.09.007

[11] Patte KA Davis CA Levitan RD Kaplan AS Carter-Major J Kennedy JL. behavioral genetic model of the mechanisms underlying the link between obesity and symptoms of ADHD. J Atten Disord. (2020) 24:1425–36. 10.1177/1087054715618793 26794671

[12] Kaisari P Dourish CT Higgs S. Attention deficit hyperactivity disorder (ADHD) and disordered eating behaviour: a systematic review and a framework for future research. Clin Psychol Rev. (2017) 53:109–21. 10.1016/j.cpr.2017.03.002 28334570

[13] Cox JH Seri S Cavanna AE. Sensory aspects of Tourette syndrome. Neurosci Biobehav Rev. (2018) 88:170–6. 10.1016/j.neubiorev.2018.03.016 29559228

[14] Ledford JR Gast DL. Feeding problems in children with autism spectrum disorders: a review. Focus Autism Dev Disabil. (2016) 21:153–66. 10.1177/1088357606021003040123371510

[15] Blissett J Bennett C Fogel A Harris G Higgs S. Parental modelling and prompting effects on acceptance of a novel fruit in 2–4-year-old children are dependent on children's food responsiveness. Br J Nutr. (2016) 115:554–64. 10.1017/S0007114515004651 26603382

[16] Farrow CV Haycraft E Blissett JM. Teaching our children when to eat: how parental feeding practices inform the development of emotional eating—a longitudinal experimental design. Am J Clin Nutr. (2015) 101:908–13. 10.3945/ajcn.114.103713 25787999

[17] Johnson SL Birch LL. Parents' and children's adiposity and eating style. Pediatrics. (1994) 94:653–61. 10.1542/peds.94.5.653 7936891

[18] Jansen PW Tharner A van der Ende J Wake M Raat H Hofman A. Feeding practices and child weight: is the association bidirectional in preschool children? Am J Clin Nutr. (2014) 100:1329–36. 10.3945/ajcn.114.088922 25332330

[19] Wolstenholme H Kelly C Hennessy M Heary C. Childhood fussy/picky eating behaviours: a systematic review and synthesis of qualitative studies. Int J Behav Nutr Phys Act. (2020) 17:2. 10.1186/s12966-019-0899-x 31900163 PMC6942299

[20] Öz S Bayhan P. An investigation of the relationship between the eating behaviours of children with typical development and autism spectrum disorders and parent attitudes during mealtime eating behaviours and parent attitudes during mealtime. Child Care Health Dev. (2021) 47:877–85. 10.1111/cch.12899 34273188

[21] Mohammadi MR Ahmadi N Khaleghi A Mostafavi SA Kamali K Rahgozar M. Prevalence and correlates of psychiatric disorders in a national survey of iranian children and adolescents. Iran J Psychiatry. (2019) 14:1–15. 10.18502/ijps.v14i1.418 31114613 PMC6505051

[22] Brander G Isomura K Chang Z Kuja-Halkola R Almqvist C Larsson H. Association of tourette syndrome and chronic tic disorder with metabolic and cardiovascular disorders. JAMA Neurol. (2019) 76:454–61. 10.1001/jamaneurol.2018.4279 30640363 PMC6459125

[23] Tic Disorders Are Associated With Obesity and Diabetes. Available online at: https://www.mdedge.com/neurology/article/192599/movement-disorders/tic-disorders-are-associated-obesity-and-diabetes (accessed July 12, 2022).

[24] Degrauw RS Li JZ Gilbert DL. Body mass index changes and chronic neuroleptic drug treatment for tourette syndrome. Pediatr Neurol. (2009) 41:183–6. 10.1016/j.pediatrneurol.2009.04.002 19664533

[25] Woods DW Piacentini J Himle MB Chang S. Premonitory urge for tics scale (PUTS): initial psychometric results and examination of the premonitory urge phenomenon in youths with tic disorders. J Dev Behav Pediatr. (2005) 26:397–403. 10.1097/00004703-200512000-00001 16344654

[26] Ehlers S Gillberg C Wing L A screening questionnaire for asperger syndrome and other high-functioning autism spectrum disorders in school age children. J Autism Dev Disord. (1999) 29:129–41. 10.1023/A:1023040610384 10382133

[27] Conners CK Sitarenios G Parker JDA Epstein JN. The revised conners' parent rating scale (CPRS-R): factor structure, reliability, and criterion validity. J Abnorm Child Psychol. (1998) 26:257–68. 10.1023/a:1022602400621 9700518

[28] Child Growth Foundation. Child Growth Foundation Cross Sectional Stature and Weight Reference Curves for the UK Child Growth Foundation, London (1996)

[29] Cole TJ Bellizzi MC Flegal KM Dietz WH. Establishing a standard definition for child overweight and obesity worldwide: international survey. BMJ. (2000) 320:1240. 10.1136/bmj.320.7244.1240 10797032 PMC27365

[30] Cole TJ Flegal KM Nicholls D Jackson AA. Body mass index cut offs to define thinness in children and adolescents: international survey. BMJ. (2007) 335:194. 10.1136/bmj.39238.399444.55 17591624 PMC1934447

[31] Aykutlu H Görker I. Disruptive eating behavior and obesity in drug naïve children diagnosed with attention deficit hyperactivity disorder. Anatol J Psychiatry. (2019) 2019:1. 10.5455/apd.28613

[32] Leventakou V Micali N Georgiou V Sarri K Koutra K Koinaki S. Is there an association between eating behaviour and attention-deficit/hyperactivity disorder symptoms in preschool children? J Child Psychol Psychiatry. (2016) 57:676–84. 10.1111/jcpp.12504 26706046

[33] Johnson SL Taylor-Holloway LA. Non-hispanic white and hispanic elementary school children's self-regulation of energy intake. Am J Clin Nutr. (2006) 83:1276–82. 10.1093/ajcn/83.6.1276 16762937

[34] Haycraft E Farrow C Meyer C Powell F Blissett J. Relationships between temperament and eating behaviours in young children. Appetite. (2011) 56:689–92. 10.1016/j.appet.2011.02.005 21316412

[35] Freitas A Albuquerque G Silva C Oliveira A. Appetite-related eating behaviours: an overview of assessment methods, determinants and effects on children's weight. Ann Nutr Metab. (2018) 73:19–29. 10.1159/000489824 29843129

[36] Aparicio E Canals J Arija V De Henauw S Michels N. The role of emotion regulation in childhood obesity: implications for prevention and treatment. Nutr Res Rev. (2016) 29:17–29. 10.1017/S0954422415000153 27045966

[37] Nguyen-Rodriguez ST Unger JB Spruijt-Metz D. Psychological determinants of emotional eating in adolescence. Eat Disord. (2009) 17:211–24. 10.1080/10640260902848543 19391020 PMC2859040

[38] Coffey BJ Biederman J Smoller JW Geller DA Sarin P Schwartz S. Anxiety disorders and tic severity in juveniles with tourette's disorder. J Am Acad Child Adolesc Psychiatry. (2000) 39:562–8. 10.1097/00004583-200005000-00009 10802973

[39] Kyriazi M Kalyva E Vargiami E Krikonis K Zafeiriou D. Premonitory urges and their link with tic severity in children and adolescents with tic disorders. Front Psychiatry. (2019) 10:00569. 10.3389/fpsyt.2019.00569 31474885 PMC6702331

[40] Martino D Pringsheim TM Cavanna AE Colosimo C Hartmann A Leckman JF. Systematic review of severity scales and screening instruments for tics: critique and recommendations. Mov Disord. (2017) 32:467–73. 10.1002/mds.26891 28071825 PMC5482361

[41] Yan J Yu L Wen F Wang F Liu J Cui Y. The severity of obsessive-compulsive symptoms in Tourette syndrome and its relationship with premonitory urges: a meta-analysis. Expert Rev Neurother. (2020) 20:1197–1205. 10.1080/14737175.2020.1826932 32954857

[42] Williams KE Hendy H Knecht S. Parent feeding practices and child variables associated with childhood feeding problems. J Dev Phys Disabil N Y. (2008) 20:231–42. 10.1007/s10882-007-9091-3

[43] Faith MS Kerns J. Infant and child feeding practices and childhood overweight: the role of restriction. Matern Child Nutr. (2005) 1:164–8. 10.1111/j.1740-8709.2005.00024.x 16881896 PMC6860954

[44] Hendy HM Williams KE Riegel K Paul C. Parent mealtime actions that mediate associations between children's fussy-eating and their weight and diet. Appetite. (2010) 54:191–5. 10.1016/j.appet.2009.10.006 19887094

[45] Carruth BR Ziegler PJ Gordon A Barr SI. Prevalence of picky eaters among infants and toddlers and their caregivers' decisions about offering a new food. J Am Diet Assoc. (2004) 104:57–64. 10.1016/j.jada.2003.10.024 14702019

[46] Westenhöfer J Pudel V. Failed and successful dieting: risks of restrained eating and chances of cognitive control. Int J Obes. (1996) 18:481–7.

[47] Warkentin S Mais LA Ranganath K Jansen E Carnell S. Controlling and less controlling feeding practices are differentially associated with child food intake and appetitive behaviors assessed in a school environment. Pediatr Obes. (2020) 15:e12714. 10.1111/ijpo.12714 32893452

[48] Chai LK Collins CE May C Holder C Burrows TL. Accuracy of parent-reported child height and weight and calculated body mass index compared with objectively measured anthropometrics: secondary analysis of a randomized controlled trial. J Med Internet Res. (2019) 21:e12532. 10.2196/12532 31538954 PMC6754693

[49] Pesch MH Lumeng JC. Methodological considerations for observational coding of eating and feeding behaviors in children and their families. Int J Behav Nutr Phys Act. (2017) 14:170. 10.1186/s12966-017-0619-3 29246234 PMC5732463

[50] Ludlow AK Rogers SL. Understanding the impact of diet and nutrition on symptoms of Tourette syndrome: a scoping review. J Child Health Care. (2018) 22:68–83. 10.1177/1367493517748373 29268618

[51] Pauls DL Raymond CL Robertson MM. The genetics of obsessive-compulsive disorder. Psychobiological. (1991) 1991:89–100.

[52] Zohar AH Ratzoni G Pauls DL Apter A Bleich A Kron S. An epidemiological study of obsessive-compulsive disorder and related disorders in israeli adolescents. J Am Acad Child Adolesc Psychiatry. (1992) 31:1057–61. 10.1097/00004583-199211000-00010 1429405

[53] Holzer JC McDougle CJ Boyarsky BK Price LH Goodman WK Baer L. Obsessive–compulsive disorder with and without a chronic tic disorder: a comparison of symptoms in 70 patients. Br J Psychiatry. (1994) 164:469–73. 10.1192/bjp.164.4.469 8038934

